# A Data-Driven Approach for the Diagnosis of Mechanical Systems Using Trained Subtracted Signal Spectrograms

**DOI:** 10.3390/s19051055

**Published:** 2019-03-01

**Authors:** Jiung Huh, Huan Pham Van, Soonyoung Han, Hae-Jin Choi, Seung-Kyum Choi

**Affiliations:** 1 School of Mechanical Engineering, Chung-Ang University, Seoul 06974, Korea; puraria@cau.ac.kr (J.H.); pvh134@cau.ac.kr (H.P.V.); hsy4190@cau.ac.kr (S.H.); 2 The George W. Woodruff School of Mechanical Engineering, Georgia Institute of Technology, Atlanta, GA 30332, USA

**Keywords:** prognostics and health management (PHM), industrial robot, critical information map (CIM), data-driven, non-stationary signal, smart factory, wavelet package decomposition (WPD)

## Abstract

Toward the prognostic and health management of mechanical systems, we propose and validate a novel effective, data-driven fault diagnosis method. In this method, we develop a trained subtracted spectrogram, the so called critical information map (CIM), identifying the difference between the signal spectrograms of normal and abnormal status. We believe this diagnosis process may be implemented in an autonomous manner so that an engineer employs it without expert knowledge in signal processing or mechanical analyses. Firstly, the CIM method applies sequential and autonomous procedures of time-synchronization, time frequency conversion, and spectral subtraction on raw signal. Secondly, the subtracted spectrogram is then trained to be a CIM for a specific mechanical system failure by finding out the optimal parameters and abstracted information of the spectrogram. Finally, the status of a system health can be monitored accurately by comparing the CIM with an acquired signal map in an automated and timely manner. The effectiveness of the proposed method is successfully validated by employing a diagnosis problem of six-degree-of-freedom industrial robot, which is the diagnosis of a non-stationary system with a small amount of training datasets.

## 1. Introduction

Uncertainty in engineering has been considered as a critical problem as it could result in serious financial losses or catastrophic accidents. Specifically, if a machine in a production line suddenly malfunctions, the line will stop, which leads to significant financial losses. Therefore, it is critical to predict malfunctions or the life of manufacturing machines. This requirement has resulted in active research regarding the prognostics and health management of machinery with the benefit of the fourth industrial revolution from the significant progress in data science, computer performance, and communication.

For the health monitoring or diagnosis of a machine, we need sensors to measure the status of the machine, including sensors to measure temperature [1], pressure [2], volume pressure [3,4], and acceleration [5,6,7]. Among these sensors, acoustic emission sensors and accelerometers are the most commonly used for monitoring machines as they can provide instantaneously the status of a machine with high data sampling frequencies, which is not possible with temperature or pressure sensors. In other words, we can obtain large amounts of information over a relatively short measurement time. Accelerometer signals that measure structural vibration are especially useful in monitoring rotary machines, such as gear sets and bearings, as their frequencies are closely related to the frequency of the mechanical mesh and the machine structure [8,9]. In this study, we investigate the techniques for monitoring machinery with the acceleration signals.

The approaches for the fault diagnosis are categorized into model-based, signal-based, knowledge-based, hybrid-based [10], and active approaches [11,12]. Among the five approaches, model-based, signal-based and knowledge-based approaches are mostly applied in the machine fault diagnosis. Model-based methods employ specific dynamic models or theories which simulate the real condition. Luo [13] proposed a shape-independent method to model different kinds of tooth spalls and validated by finite element analyses. Endo [14] diagnosed spalls and cracks on the gear tooth based on simulated signals. Park [15] diagnosed gear faults in planetary gears using transmission errors simulated from a dynamic model.

Signal-based methods use the aforementioned signals with prior knowledge. Lebold [16] reported a signal processing procedure to extract features from the vibration signal for monitoring a gear set. The first step in this method was extracting statistical parameters, such as root mean square, kurtosis, and skewness, from raw vibrational signal that could include important information regarding mechanical faults. The second step was extracting features from the cyclic signal from which noise is removed by the time synchronous averaging (TSA) technique [8]. Additional signal features could be obtained from the residual signal remaining after removing the gear mesh frequency, the difference signal obtained by further removing the sideband of the mesh frequency, or the band-pass mesh signal, which is the band frequency signal extracted from the TSA signal. He et al. [17] employed bearing characteristic frequencies as the representative features for the analysis of bearing faults. The bearing characteristic frequencies are the metrics of frequency densities related to a specific part of a bearing. Statistical parameters, such as kurtosis, are also used to measure degradation and the degree of bearing defects. 

The model-based and signal-based methods are commonly employed for the diagnosis of gear sets and bearings. However, these approaches are available only when the signal is measured in a stationary status. It is also virtually impossible for engineers to apply the approaches to a system of which structure is complex and dynamic characteristics, such as operational frequency of the gear sets and bearings, is not sufficient. In order to overcome these limitations and meet the demands of diagnoses of various system types, knowledge-based methods, also called data-driven methods, have been proposed. A considerable number of research works [18,19,20,21] based on the approach have been carried out recently. In the knowledge-based methods, professional information or knowledge of the systems are not necessary. Instead, a machine-learning algorithm recognizes patterns of each class and diagnoses the systems by themselves. However, it requires a large amount of historical data to find important information for the fault diagnoses [22]. In order to overcome the limitations of the three different methods, we propose a new fault diagnosis method called a critical information map (CIM), that works with (1) relatively small amounts of data, (2) non-stationary signals, and (3) unknown system structures and dynamics as discussed in this study. 

The details of our idea are presented in the following sections: Section 2 explains pre-processing processes, which apply to the raw signals, include data synchronization, time frequency representation (TFR) transformation, and spectral subtraction. Section 3 includes an optimization process that addresses the important parameters to achieve critical information map. A case study of data acquiring from manipulator is introduced and the CIM method is validated in Section 4. Another case study based on the data from the National Aeronautics and Space Administration (NASA) repository has been employed to validate the usefulness of the CIM, comparing performance with other methods in Section 5. Finally, in Section 6, we discuss the validation results and the usefulness of this technique in a prognostics and health management (PHM) problem. 

## 2. Critical Information Map (CIM): Pre-Processing

In this study, we propose a TFR-based CIM that includes the information of the locations within the TFR spectrograms that shows clear (or large) difference between normal and fault conditions. An engineer can diagnose the system fault by identifying differences of the parameters at the specific regions of the CIM of a system of interest. Using this approach, we believe that detailed structural or dynamic knowledge of a system is not necessary since we employ TFR-based spectrograms. In addition, the proposed approach is applicable to the non-stationary signal-based diagnoses as we preserve and make use of the domain of the TFRs. Finally, this approach requires relatively small amounts of training data compared to that required by the TFR and convolutional neural network (CNN) approaches.

**Figure 1:**
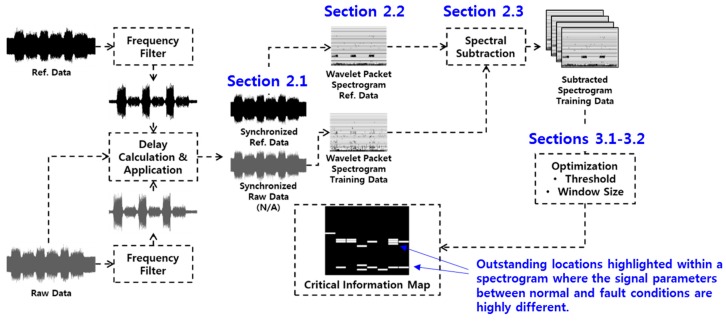
The process of building the critical information map (CIM).

The overall procedure of creating a CIM with raw measured vibrational signals is illustrated in Figure 1. It comprises three pre-processes: data synchronization that is discussed in Section 2.1, wavelet packet transformation discussed in Section 2.2, and spectral subtraction explained in Section 2.3. Without the pre-processing steps, the following step (i.e., optimization process) cannot be converged. The final optimization process for creating CIMs is discussed in Section 3. 

### 2.1. Data Synchronization

Measured data typically includes a time delay that needs to be eliminated by synchronization. In this study, we employ cross-correlation for the time synchronization. Before the synchronization, we first extract non-stationary movement signals of interest using a band-pass filter as other unrelated signals become noise and hinder the time synchronization.

Cross-correlation is typically used to synchronize two different time-series signals. The conformity degree between the two signals is defined as $\left(f\times g\right)\left(\tau \right)=\;\int_{-\infty }^{\infty }f\left(t\right)g\left(t+\tau \right)dt$ where *f* is the original function, g is the filtering function, and τ is the time delay between the two signals [23]. We determine the time delay ($\tau_{max})\;$that maximizes the cross-correlation, (f×g)(τ), which is $\tau_{max}=Argmax\left\{\left(f\times g\right)\left(\tau \right)\right\}$. The raw signal is then synchronized with the original signal for comparison by applying the $\tau_{max}$.

### 2.2. Time Frequency Representation (TFR) Transformation

For developing CIM, we convert raw signals to TFRs (i.e., two-dimensional data plots) for the effective representation of the signal information for humans as well as computers. Short-time Fourier transform (STFT), wavelet transform (WT), and wavelet packet decomposition (WPD) are the linear methods commonly used for the transformation [24]. The primary differences between the three methods are the form of the filter and the shapes of the spectrum tile (i.e., window) delivered from the filter. 

**Figure 2:**
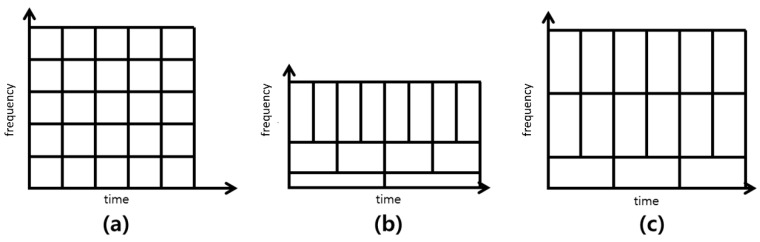
Window shapes: (**a**) short-time Fourier transform (STFT); (**b**) wavelet transform (WT); (**c**) wavelet packet decomposition (WPD).

In the STFT method, the size of the windows is pre-determined and identical for all frequency and time domains, as shown in Figure 2a. In addition, one frequency density value is assigned for each window. The pre-determination of the window size can, at times, increase the degree of uncertainty in the high- and low-frequency domains of the transformed diagrams [25]. Verstraete [26] reported that CNN models that were trained based on the two-dimensional image data transformed using the STFT method exhibited low algorithm reliability. The WT method overcomes this limitation by varying the size of the window of each time and frequency domain, as shown in Figure 2b. However, in WT method, the resolution of the time and frequency domains are determined by the number of windows, which makes the window sizes uneven in the domain. Additionally, WT decomposes only the low frequency component at subsequent levels where as WPD decomposes both low- and high-frequency components at each level. WT is not desirable for creating a CIM for fault identification where signal contains high frequency information. The WPD method addresses the limitations of the two methods.

As can be seen in Figure 2c, the WPD method assigns a different filter at each divided domain, which provides a high degree of freedom in setting the size of tiles. Compared to the STFT method that uses only one filter, we can obtain more reliable frequency density values with the WPD method. In this study, we employ WPD as the TFR method for creating the CIM. In WPD, the kernel function of the wavelet packet to decompose the signal to several frequencies is:

(1) $$W_{j,k}^{n}\left(t\right)=2^{\frac{j}{2}}W^{n}\left(2^{j}t-k\right),$$

Equation (1) includes three positive integer constants: j is the index scale, k is the translation operation, and n is the modulation parameter or oscillation parameter. The first and second wavelet packet functions need to be predefined, as shown in Equations (2) and (3), which are known as the usual scaling function and mother function, respectively.

(2) $$W_{0,0}^{0}\left(t\right)=\phi \left(t\right),$$

(3) $$W_{0,0}^{1}\left(t\right)=\psi \left(t\right),$$

additional functions may be created as follows:

(4) $$W_{0,0}^{2n}\left(t\right)=\sqrt{2}\underset{k}{\sum }h\left(k\right)W_{1,k}^{n}\left(2t-k\right),$$

(5) $$W_{0,0}^{2n+1}\left(t\right)=\sqrt{2}\underset{k}{\sum }g\left(k\right)W_{1,k}^{n}\left(2t-k\right),$$

where h(k) and g(k) are the quadrature mirror filters, which are orthogonal to each other. The wavelet packet coefficient in a *j, n, k* state that represents the density of a filter is:

(6) $$\omega_{j,n,k}\left(t\right)=\langle f,W_{j,k}^{n}\rangle =\int f\left(t\right)W_{j,k}^{n}\left(t\right)dt\;,$$

where f(t) is the time signal that will be analyzed. The values of the wavelet packet coefficients that will be used for developing the CIMs are displayed in the WPD spectrogram in Figure 2c. The details of the WPD are beyond the scope of this study, and can be found in [27] 

### 2.3. Spectral Subtraction 

In this study, we use the spectral subtraction technique to determine the critical area on the WPD by which we can screen fault conditions from the samples. The spectral subtraction technique has been employed by acoustics engineers to filter out unwanted noise. The basic principle is to extract signals in the domain where speech signals do not exist, and then using the extracted signal to enhance the voices.

In the spectral subtraction, the modified signal spectrum is as follows:

(7) $$P_{s}^{\prime }\left(w\right)=\;\{\begin{array}{c} P_{s}\left(w\right)-\;P_{n}\left(w\right),\;\;\;\;if\;P_{s}\left(w\right)-\;P_{n}\left(w\right)>0\\ 0,\;\;\;\;\;\;\;\;\;\;\;\;\;\;\;\;\;\;\;\;\;\;\;\;\;\;\;otherwise \end{array},$$

where$\;P_{s}\left(w\right)$ is the spectrum with speech and noise, and $P_{n}\left(w\right)$ is the noise spectrum without speech. Berouti et al. [28] obtained speech signals without noise by the Fourier inversion of $P_{s}^{\prime }\left(w\right)$. Denda [29] employed spectral subtraction based on a wavelet transformed spectrum, which is represented as follows:

(8) $$\left|\;\hat{X}\left(b,a\right)\right|=\left|Y_{s}\left(b,a\right)\right|-\;\alpha \left|\bar{N_{n}\left(b,a\right)}\right|,$$

where $\left|\hat{X}\left(b,a\right)\right|$ is the wavelet spectrum of enhanced speech, $\left|Y_{s}\left(b,a\right)\right|$ is the wavelet spectrum of the observed signal, and $\left|\bar{N_{n}\left(b,a\right)}\right|$ the wavelet spectrum of noise. In addition, α is the reduction factor, and *a* and *b* are the filter location and scale parameters in the wavelet transformation spectrum, respectively. El Bouchikhi [30] assumed the spectrum domain without speech as the signal from normal bearings and the spectrum domain with speech as the signal from faulty bearings. After the spectral subtraction, El Bouchikhi obtained improved diagnosis results.

In this study, we performed the spectral subtraction in Equation (8) based on the spectrogram obtained by WPD to identify the critical region of the spectrogram for fault identification. We subtracted the signal spectrum of the normal condition from that of the abnormal condition, which indicates that specific features of the normal condition data are removed. In this study, normal-reference (N-R) indicates the spectrogram obtained by subtracting the reference signal from the normal condition signal. A normal dataset is randomly selected as the reference signal for this spectral subtraction to fix a ground value of the spectral coefficient. Abnormal-reference (A-R) is then the spectrogram derived by subtracting the reference signal from the abnormal one. We mutually subtract between the spectrums of N-R and A-R to obtain the degree of random error, such as measurement error. We discuss the technique to deal with the random error for robust decision-making in diagnosis in Section 3. 

## 3. Critical Information Map: Optimization

### 3.1. CIM Optimization (Training) Parameters

**Figure 3:**
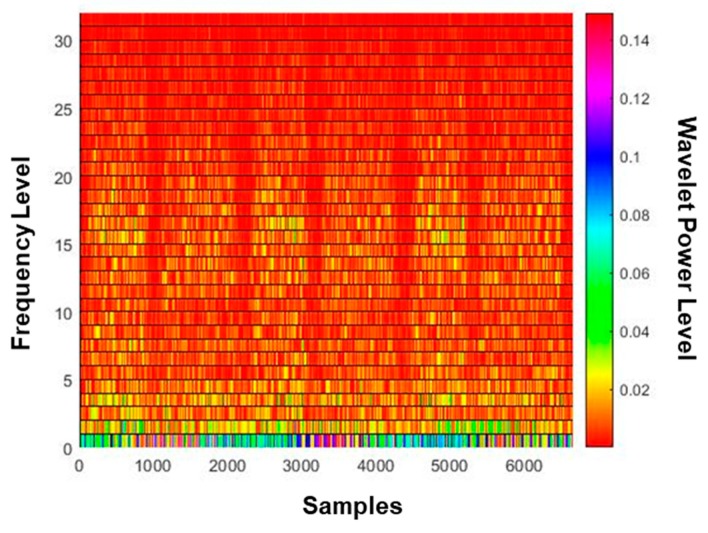
Example of subtracted spectrogram.

For developing the CIM, we need to identify the parameters that determine the location and size of important regions for each fault condition. Figure 3 is an example of a spectrogram (i.e., map) obtained after the spectral subtraction process discussed in Section 2.3. The resolution of the map may be determined by setting the number of windows (i.e., tiles) in the time domain, but the resolution in the frequency domain is fixed because the variation of the frequency domains increases the non-linearity by changing the number of samples in the time domain. A number of density values may exist in each window. There could be specific windows whose density values for normal and fault conditions are significantly different. These windows may be beneficial for identifying a fault condition. If we know the location of these windows, we can compare the values of those specific windows only. This could reduce the noise for fault diagnosis. Therefore, we need to create an optimal diagnosis map that includes the location and the threshold value of important windows for each fault condition.

In order to formulate an optimization problem for creating the CIM, we first define three design variables to determine. The first variable ($x_{1}$) is the number of windows in the time domain that also determines the size of windows (or resolution). The second variable ($x_{2}$) is the threshold value to be an outstanding parameter in a window. If a density parameter is greater than $x_{2}$, it is then an outstanding parameter. The last variable ($x_{3}$) is the threshold of the number of outstanding parameters in a window. If the number of outstanding parameters in a window is greater than $x_{3}$, then the window becomes an important window to be observed for diagnosis.

**Figure 4:**
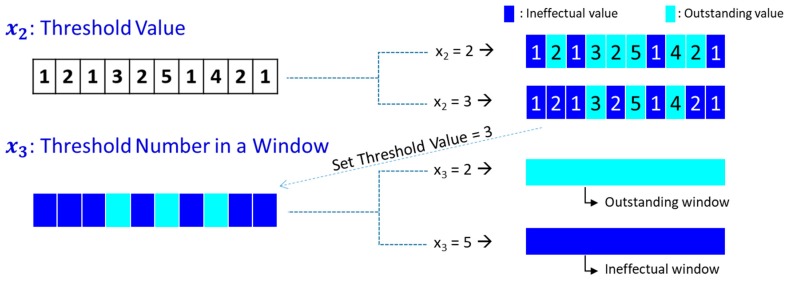
Example of design variables: $x_{2}\;$= threshold value for parameter to be outstanding, and $x_{3}\;$= threshold number of outstanding parameters for a window to be outstanding.

A simple example to better understanding the definition of the variables, $x_{2}$ and $x_{3}$, is shown in Figure 4. The window in this example comprises 10 parameters (i.e., cells) with the assigned values as shown in the figure. If $x_{2}=2$, then the outstanding parameters are the parameters of which values are greater than or equal to 2. In this case, the number of outstanding parameters in the window is 6. If $x_{2}=3$, then the number of outstanding parameters in the window becomes 3. If $x_{2}=3$ and $x_{3}=2$, then the window is outstanding. However, if $x_{2}=2$ and $x_{3}=5$, then the window is not outstanding.

### 3.2. Optimization Problem Formulation

**Figure 5:**
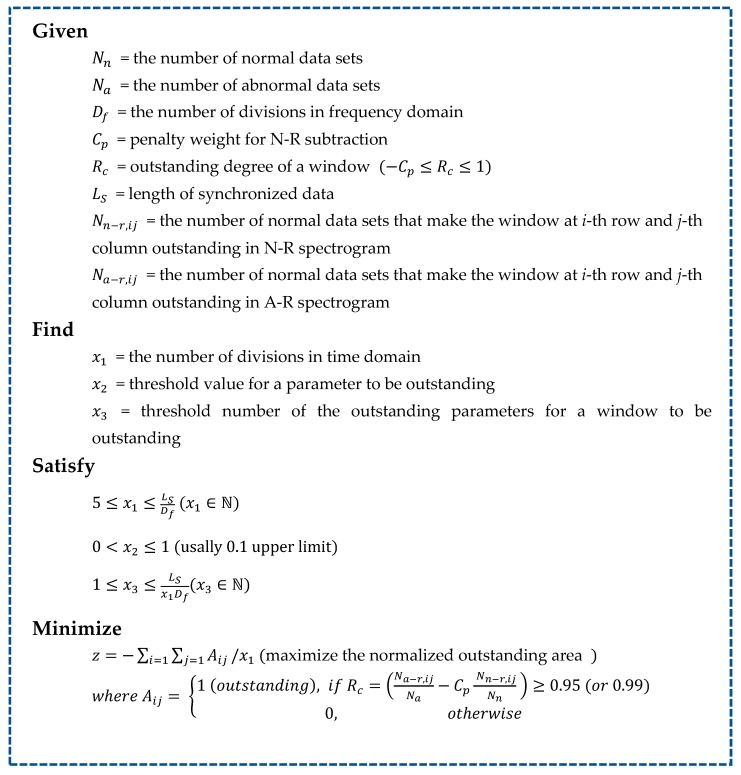
Problem formulation of CIM optimization.

Figure 5 shows the optimization problem formulation [31] to build the CIM. Our goal in this problem is to minimize the loss of important information. Therefore, the objective function is to maximize the normalized outstanding area (i.e., important regions), which is the number of outstanding windows normalized by the division number in the time domain. As mentioned in Section 2.3, it is important to identify the area where N-R is dominant, which will result in creating a map highly sensitive to random noise (or measurement error). For this reason, we employed the outstanding degree of a window as $R_{c}=N_{a-r,ij}/N_{a}-C_{p}N_{n-r,ij}/N_{n}$ where $C_{p}N_{a-r,ij}/N_{a}$ is the term that represents the disturbing errors. It is recommended that the$\;R_{c}$ of a window be close to one (i.e., greater than 0.95 or 0.99) to be selected as an outstanding window. 

When deciding on the status of a window, if the number of N-R datasets (i.e., $N_{n-r,ij}$) that make the window outstanding is significantly greater than the number of A-R datasets (i.e., $N_{a-r,ij}$) that make the window outstanding, then the window can be set as an outstanding window. We can obtain the normalized outstanding area by the number of outstanding windows divided by the number of divisions in the time domain, $x_{1}$. By achieving the optimal solution of this problem, we can obtain the optimal resolution of the CIM as well as the list of outstanding windows. For the diagnosis of a new observation based on the CIM, we merely need to calculate the number of outstanding windows from the observed signal and compare it with the outstanding window list of the CIM. 

## 4. Case Study 1: Diagnosis of Input Gear Problems of Six-Degree-of-Freedom Manipulator

### 4.1. Data Acquisition Process

**Figure 6:**
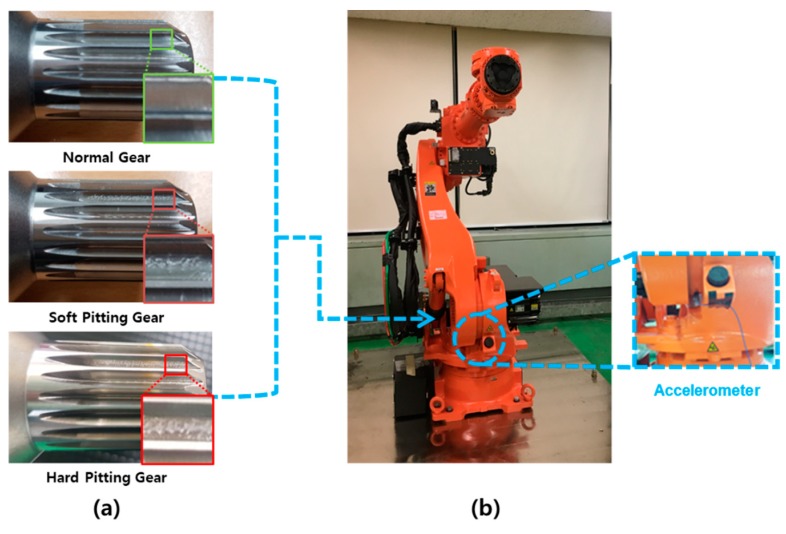
(**a**) Three different types of input gear at second joint, and (**b**) HS-180 manipulator and position of accelerometer.

We apply the CIM-based diagnosis method proposed in Section 2 and Section 3 to the diagnosis of a six-degree-of-freedom manipulator. This industrial robust system is a suitable example for validating the proposed method as it comprises numerous components, including arms, motors, belts, bearings, and gear sets. The signal observed from this system is, therefore, typically complex and non-stationary. The manipulator employed in this case study is the HS-180S, manufactured by Hyundai Robotics Inc in Daegu, Korea. (https://www.hyundai-robotics.com/), as shown in Figure 6b.

The analysis target is the second joint of the manipulator shown in Figure 6b. As can be seen in the figure, an accelerometer is attached on top of the joint housing to measure the radial vibration [32]. In this case study, we assumed that the input gear of the second joint could have pitting defects during the operation, as shown in Figure 6a. The top photo is the normal input gear, the middle one is the gear with soft pitting, and the bottom one is the gear with hard pitting. The pitting defects were intentionally created with a grinder for the purposes of testing. Soft and hard pitting defects depend on the amount of scratching on the surface of the gear tooth. 

**Figure 7:**
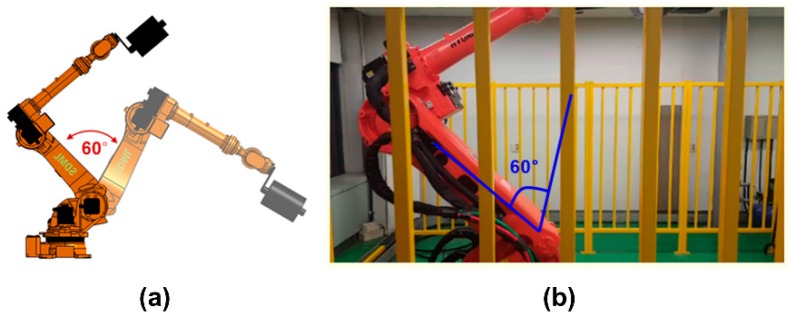
Data acquiring motion of robot shown in (**a**) a graphical image and (**b**) its photograph.

The motion of the manipulator for acquiring signal data is rotating the upper and lower rams three times in 60° around the section joint, as shown in Figure 7. This action was repeated 300 times to collect 900 sets of accelerometer data (300 normal dataset, 300 hard-pitting dataset, 300 soft-pitting dataset), which are sufficient for developing and validating our approach. In other words, we could have 300 normal datasets and 600 abnormal datasets, which include hard pitting and soft pitting faults. The time duration is 20 s for the three rotations, and the sampling rate is 12.8 kHz. The primary data acquisition specifications are presented in Table 1.

### 4.2. Data Synchronization

**Figure 8:**
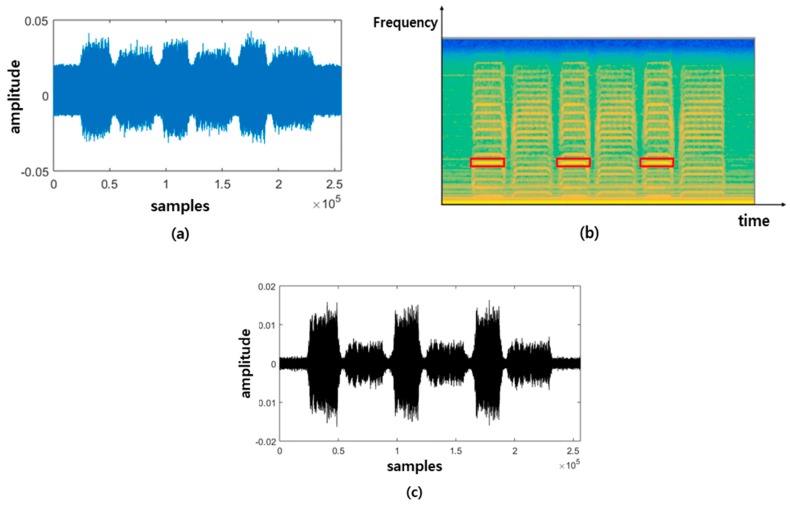
(**a**) Set of acquired raw signal data, (**b**) spectrogram of raw data, and (**c**) filtered data.

**Figure 9:**
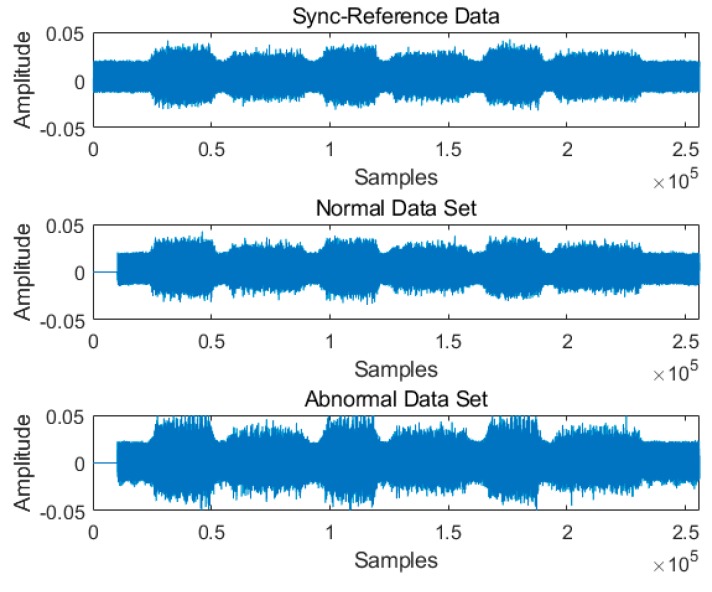
Time synchronization of raw signal data.

Figure 8a is the raw signal data acquired from the accelerometer with the normal input gear shown in Figure 6a. The signal with the larger amplitude and the shorter time duration is acquired during the downward movement, and the signal with the smaller amplitude and the longer time duration during the upward movement. In order to determine the frequency bandwidth useful for data synchronization, we draw a continuous spectrogram of the raw signal, as shown in Figure 8b, and extract the signal of the bandwidth in the rectangular area, which is the most distinct after the band-pass filter, as shown in Figure 8c. We determine the time delay from the filtered signals and apply it to the raw signal for time synchronization. An example of the data synchronization results is shown in Figure 9.

### 4.3. Wavelet Packet Decomposition (WPD) Spectrogram and Spectral Subtraction

**Figure 10:**
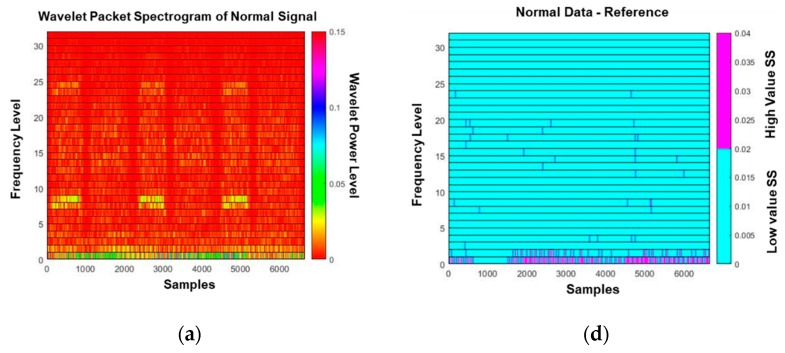

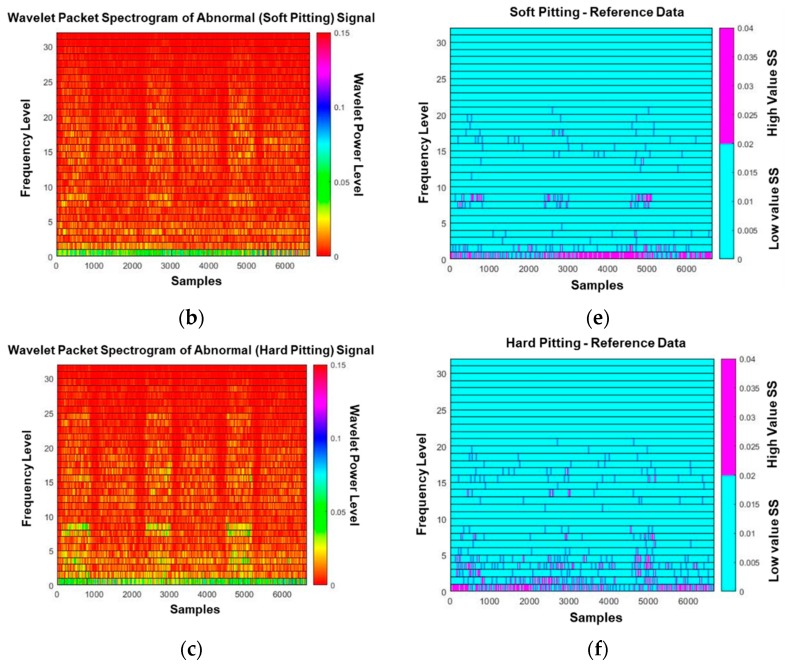
Wavelet packet spectrograms of (**a**) a normal signal, (**b**) an abnormal (soft-pitting) signal, and (**c**) another abnormal (hard-pitting) signal; (**d**) N-R spectrogram, (**e**) A-R (S-R) spectrogram, (**f**) A-R (H-R) spectrogram.

The synchronized signals are transformed into spectrograms by WPD. Each image of the spectrograms includes 6656 parameters in the time domain (i.e., horizontal direction) and 32 parameters in the frequency domain (i.e., vertical direction). Figure 10a–c shows the wavelet packet spectrogram of normal signal, soft-pitting signal and hard-pitting signal, respectively. The distinction between these spectrograms are not clear. As shown in Figure 10b, the area with purple color bars in the N-R spectrogram indicate the regions of significant differences in the parameter values between the normal and reference spectrograms, where the reference spectrogram is an instance of normal spectrograms. Compared with Figure 10d,e, we can observe that the size of the purple area of the S-R spectrogram is greater than that of the N-R spectrogram. This indicates that the difference between the abnormal (soft pitting) and reference spectrograms is greater than that between the normal and reference spectrograms. To distinguish the abnormal signals from the normal signal, we need only focus on the purple area highlighted in Figure 10e and not in Figure 10d. A similar phenomenon might be observed when we compare the H-R spectrogram in Figure 10f with other subtracted spectrograms. It is a reasonable assumption that the purple area highlighted in Figure 10d is from the random noise as we randomly select a normal signal as the reference signal. This screening process is essential for making a robust decision insensitive to the random errors in measured signals for the diagnosis of abnormal conditions. This process will be performed in the CIM optimization process discussed in Section 4.4. 

### 4.4. Optimization for Creating CIMs

**Figure 11:**
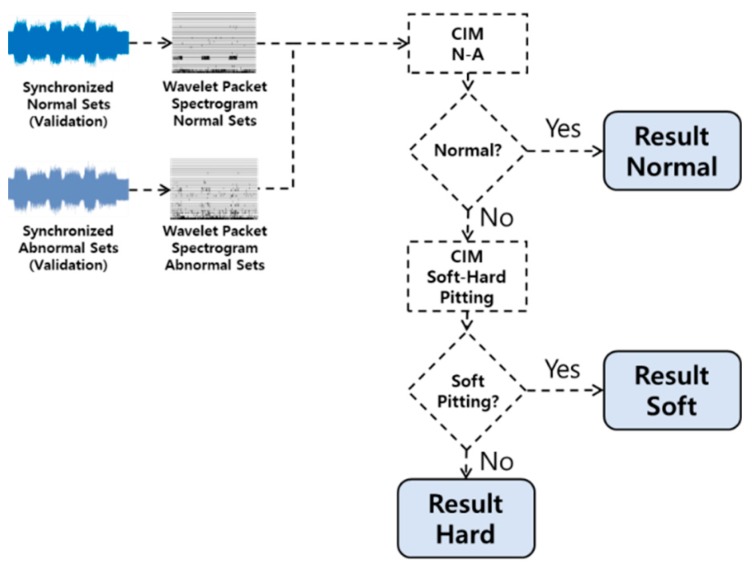
Flow chart of classifier.

In this case study, we classify the defect conditions (i.e., soft and hard pitting) of the gear, as shown in Figure 11. We first classify an input gear into a normal or abnormal condition, and then classify the abnormal gears into soft pitting or hard pitting defects. Therefore, we develop two CIMs that are for classifying normal, or abnormal conditions with soft pitting or hard pitting. Of the 900 datasets measured in Section 4.1, we use only 5% of them for developing the two CIMs, which are 15 datasets of normal, soft pitting, and hard pitting. The rest of the datasets (i.e., 855 sets) are reserved for validation in Section 4.5. The datasets for developing the CIM were pre-processed following the steps of time synchronization, transformation into WPD spectrograms, and spectrogram subtraction, as described in Section 4.2 and Section 4.3.

For the CIM optimization shown in Figure 5, parameter $C_{p}\;$ needs to be specified by users. As the values of those parameters increase, outstanding windows (OWs) are selected in a conservative manner. Typically, $C_{p}$ is set as more than one for reducing the sensitivity random errors. In this formulation, we conservatively set $C_{p}=1.5$ and select the OWs of which $R_{c}\geq 0.95$. The optimal designs found for the two CIMs is presented in Table 2. Hard-reference (H-R) is the spectrogram obtained by subtracting the reference signal from the hard-pitting signal. S-R (soft-reference) stands for the spectrogram when we do the same with the soft-pitting signal.

**Figure 12:**
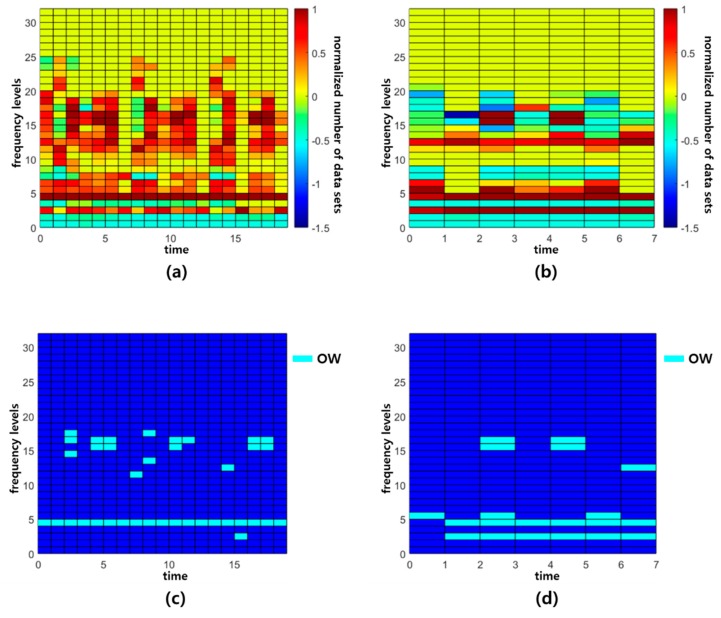
(**a**) CIM for N-R vs. A-R, (**b**) CIM for S-R vs. H-R, (**c**) selected outstanding windows (OWs) ($R_{c}$ ≥ 0.95) in CIM for N-R vs. A-R, and (**d**) selected OWs ($R_{c}$ ≥ 0.95) of CIM for S-R vs. H-R.

Figure 12a,b shows the two CIMs colored with the outstanding degree $R_{c}$ of each window highlighted. If the color of a window is red (i.e., close to one), then the window could be an OW. The windows in the figures could include a large number of SS (spectral subtraction) parameters. In these CIMs, 350 and 951 SS parameters are included in each window of Figure 12a,b, respectively. Figure 12c,d shows the OWs (i.e., highlighted windows) of which$\;R_{c}\geq 0.95$. The number of OWs in the CIM for N-R vs. A-R shown in Figure 12c is 38 and that of the CIM for S-R vs. H-R shown in Figure 12d is 20.

**Figure 13:**
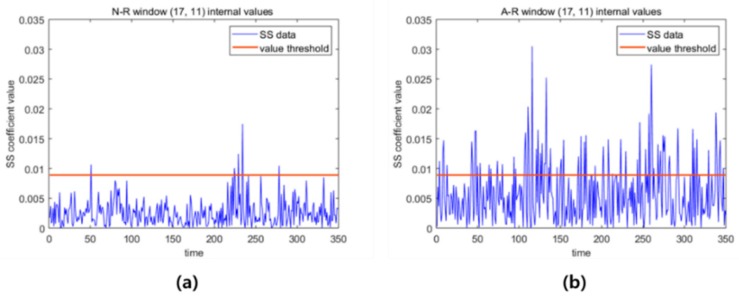
(**a**) N-R window (17, 11) SS coefficients of a training dataset, (**b**) A-R window (17, 11) SS coefficients of a training dataset.

We focus on a specific window as an example to further elaborate details of the classification based on the CIM. Figure 13 shows the SS coefficients included in the window at the 17-th frequency level and 11-th time domain. Figure 13a shows the values of 350 coefficients in the window of an N-R subtracted spectrogram obtained from a normal signal, and Figure 13b shows the same values in the same window of an A-R subtracted spectrogram from an abnormal signal. In Figure 13a, only a few SS coefficients (fewer than 10 coefficients in this example) are above the threshold based on the optimized threshold value for a parameter to be outstanding (0.0089). However, approximately half of the SS coefficients in Figure 13b are above the threshold, which is the reason why the window is selected as OW. We may classify a new observed signal data by investigating the number of windows in the O-R subtracted spectrogram that are outstanding among the OWs in the CIM. We discuss these validation results in Section 4.5. 

### 4.5. Validation Results

**Figure 14:**
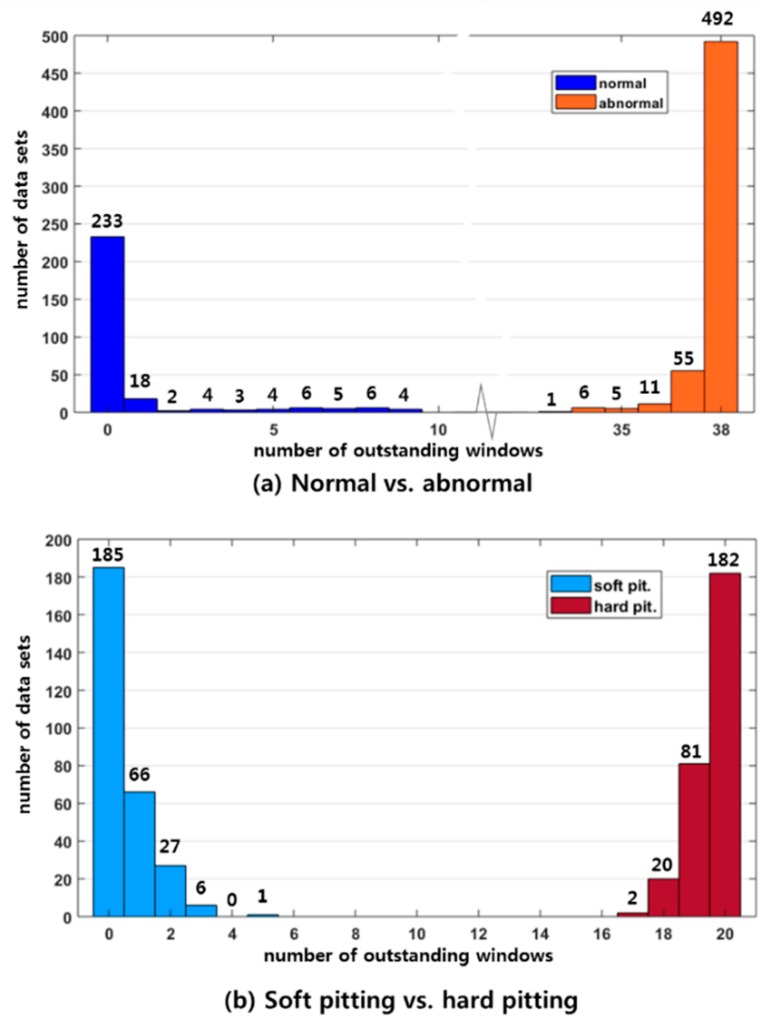
Number of OWs for classification: (**a**) normal vs. abnormal gears, and (**b**) soft pitting vs. hard pitting.

We validated the usefulness of our CIM-based classification based on the 855 datasets that were not used for developing the CIMs. Of these, one third (i.e., 285 sets) are normal, one third soft pitting, and one third hard pitting. The classification results using the CIM for N-R vs. A-R are shown in Figure 14a. The bulk of the normal datasets include no OWs among the 38 OWs in the CIM. The maximum number of OWs of normal data is nine. On the other hand, the bulk of the abnormal data (i.e., soft or hard pitting) highlights all OWs of the CIM. The minimum number of OWs of abnormal data is 33. With the reference OW number as 19, we can classify normal vs. abnormal gears with 100% accuracy. Similarly, we further classify the types of abnormal gears using the second CIM for S-R vs. H-R. The two groups of OW numbers can be clearly identified, as shown in Figure 14b. By setting the reference OW number as 10, we can also classify soft or hard pitting with 100% accuracy. This result is obtained with only 5% (15 samples of each type) of the total samples. Moreover, the computation time for the preprocessing of signals and making a decision is less than one second using a personal computer with an Intel^®^ i7 processor manufactured by LENOVO in Hong Kong, China. We believe that this is a promising result.

## 5. Case Study 2: Bearing Data Set from National Aeronautics and Space Administration (NASA) Repository

### 5.1. Bearing Data Set

The data set used in this case study is obtained from the shared website of the Prognostic Data Repository of NASA (https://ti.arc.nasa.gov/tech/dash/groups/pcoe/prognostic-data-repository/) and was often employed in many studies for the purpose of method validation. Four bearings installed in a rotating system were investigated in this study. The shaft was rotating in a constant speed (2000 revoluion per minute) by alternating current motor. A radial load of 6000 pound were applied to the shaft and bearings by a spring mechanism [33]. The data used in this case study were acquired from an accelerometer installed in one of the bearings, which had been found to be defective after the continuous run for 34 days. Each data set has 20,480 sampling points with a sampling rate of 20 kHz. The vibration signals were recorded every 10 min. Signal data were continuously collected for 34 days. The further details regarding experiment setup and data acquisition conditions might be found in [34]. In order to validate the performance of CIM, we employed the same case study that Eren did in [35], where diagnosis performances of four different techniques were compared using this NASA bearing data.

### 5.2. CIM and Comparison Results

**Figure 15:**
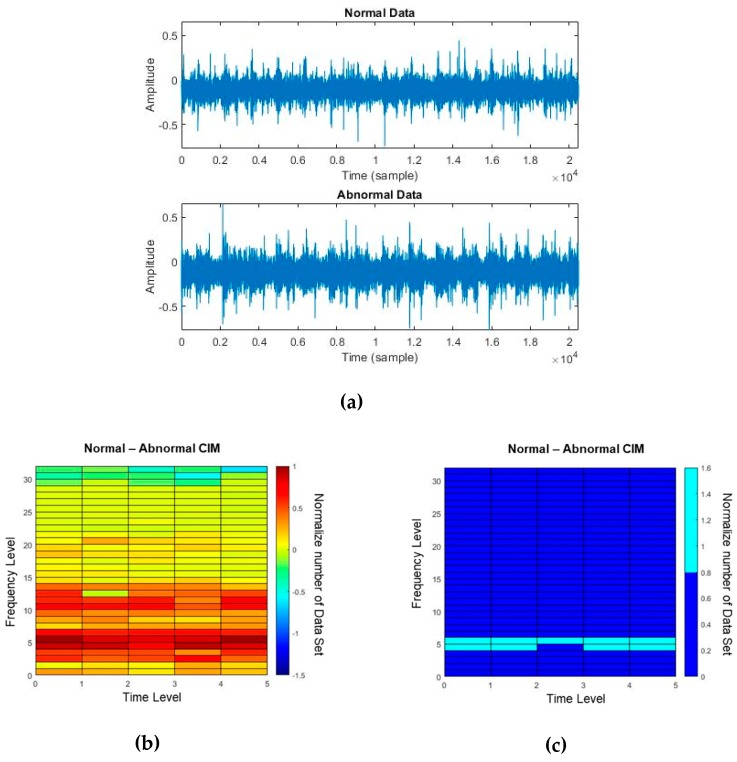
(**a**) Normal and abnormal data in time series, (**b**) CIM for N-R vs. A-R, (**c**) selected OWs ($R_{c}$ ≥ 0.8) in CIM for N-R vs. A-R.

**Figure 16:**
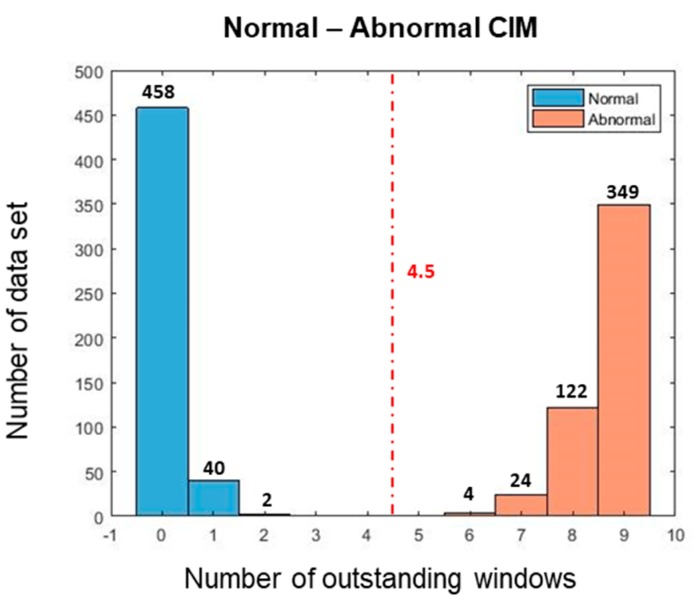
Number of OWs for classification in bearing case: normal vs. abnormal.

Table 3 shows the optimal design variables achieved after optimizing (i.e., training CIM). For the training, we used only 60 normal and 60 abnormal data sets in this case study; whereas Eren used a total of 260 runs are used in both normal and abnormal cases [34]. Figure 15a shows the raw signals of normal and abnormal conditions. Figure 15b shows the CIM obtained after the training process and Figure 15c the nine OWs selected for classifying normal vs. abnormal conditions. Since the number of OWs of the CIM is 9, we can divide normal vs. abnormal by half of 9, which is 4.5. Figure 16 shows that 500 validation data sets can be clearly classified with borderline. 

Table 4 is a comparison of confusion matrixes between the CIM approach and 1D CNN approach [35], where both of approaches used new 500 test data sets for each condition. The CIM approach achieved better classification with zero false negative and zero false positive cases while 1D CNN results include 28 false positives and one false negative. 

The standard performances, accuracy, sensitivity, specificity, and precision, of the five different methods are presented in Table 5. Accuracy, the percentage of patients correctly qualified, is (TP + TN)/(TP + TN + FP + FN). Sensitivity, the probability of a positive test result among those having the target condition, is TP/(TP + TN). Specificity, the probability of a negative test result among those without the target condition, is TN/(TN + FP). Precision, the so called positive prediction value or probability of having the target condition given a positive test result, is TP/(TP + FP). The details of the definition can be found in [36]. 

In Table 5, we compare the performance of the CIM with those of other approaches, one dimensional convolution neural network (1D CNN), multilayer perceptron (MLP), radial basis function networks (RBFN), and support vector machines (SVM). The performance data of these methods can be found in [35]. The proposed CIM approach has outstanding performance with 100% for all criteria with the smaller amount of training sets; whereas others’ performance ranged from 90% to 100%. The comparisons demonstrated that the proposed method is a promising solution when applied to the complex rotary system.

## 6. Discussion and Closure

We identified the following observations from the two examples for the validation of the proposed method. Firstly, the proposed CIM approach requires the smaller amount of training data. In the first case study, we used only 45 datasets out of 900 samples, which is only 5% of the data, for developing the CIM. The classification results with the two developed (or trained) CIMs exhibited 100% prediction accuracy, which is an outstanding result. In the second case study, the number of training data sets are 60 out of 560 in total. The results of the case study are also 100% of prediction accuracy, which cannot be achieved by other methods with much large training data. 

In addition, the CIM approach for identifying the mechanical faults could be fully automated without expert knowledge of the system being required, which is similar to the current methods, however, they need a large amount of training data. Once a signal is observed, we could accurately identify the status of a system by pre-processing (i.e., time-synchronization, TFR transformation, and spectral subtraction) and make a decision based on optimized CIM in an automated and timely manner.

The validation examples are both for non-stationary and stationary systems. As the robot arm moves upward and downward, two different types of signals, with a time gap, are obtained from the sensor. Moreover, the operating frequencies of the two movements are different as their velocities differ. In this case, the extracted signals of a frequency would be mixed with redundant data, which will increase the signal-to-noise ratio. However, the CIM approach uses TFRs and searches for OWs, which is uniquely beneficial among many different signal frequencies and time domains. This method will efficiently capture the critical information of the two completely different robot arm movements. The second case study, the rotating machine supported by the NASA repository, is a stationary system, of which classification result using the CIM approach is also very accurate with 100%. 

From the above observations, we believe that the CIM-based diagnosis approach proposed in this study is functional with a small amount of training data for the diagnosis of non-stationary and stationary systems in an automated manner without expert knowledge for extracting important signal features. However, the proposed CIM approach is designed for the diagnosis of fast dynamic systems. It may not suit slow dynamic systems, such as reactors. In addition, we need further research, such as a hybrid approach using CIM and dynamic analyses, to apply this technique to prognostics. 

## Figures and Tables

**Table 1:** Data acquisition specifications.

Features	Properties
Sensitivity of Sensor	10 mV/g
Sampling Frequency	12,800 sample/s
Acquired duration	20 s
Data Length	256,000 sample points
Normal data	300 datasets
Hard pitting data	300 datasets
Soft pitting data	300 datasets

**Table 2:** Achieved optimal CIMs.

Design Variables of CIMs	CIM (N-R vs. A-R)	CIM (S-R vs. H-R)
$x_{1}$: the number of divisions in time domain	19	7
$x_{2}$: threshold value for a parameter to be outstanding	0.0089	0.0224
$x_{3}$: the minimum number of outstanding parameters for a window to be outstanding	49	9

**Table 3:** Optimum design variables-bearing case.

CIMs Design Variable	CIM (N-R vs. A-R), Bearing Case
$x_{1}$	5
$x_{2}$	0.2658
$x_{3}$	4

**Table 4:** Comparison of confusion matrices between CIM approach and 1D CNN approach.

Method		Ground Truth	
		Normal	Abnormal
**Classification result of CIM**	**Normal**	500 (TN)	0 (FP)
	**Abnormal**	0 (FN)	500 (TP)
**Classification result of 1D CNN**	**Normal**	472 (TN)	28 (FP)
	**Abnormal**	1 (FN)	499 (TP)

**Table 5:** Bearing fault detection performance of the proposed method.

Method	Fault Detection			
	Accuracy	Sensitivity	Specificity	Precision
Proposed CIM	100	100	100	100
1D CNN	97.1	99.8	94.4	94.7
FFT-MLP	95.0	100	90.0	90.9
FFT-RBFN	96.0	100	92.0	92.6
FFT-SVM	94.5	99.0	90.0	90.8

## Nomenclature

t	real time in raw signal
τ	time delay of signal
$W_{j,k}^{n}\left(t\right)$	wavelet packet kernel function
j	wavelet packet index scale
k	wavelet packet translation operation
n	wavelet packet modulation parameter
ϕ	wavelet packet first mother function
ψ	wavelet packet second mother function
h(k)	wavelet packet quadrature mirror filter
g(k)	wavelet packet quadrature mirror filter
$\omega_{j,n,k}\left(t\right)$	wavelet packet coefficient
$P_{s}^{\prime }\left(w\right)$	signal spectrum denoised by spectral subtraction
$P_{s}\left(w\right)$	raw signal spectrum
$P_{n}\left(w\right)$	noise signal spectrum
$\hat{X}\left(b,a\right)$	wavelet spectrum denoised by spectral subtraction
$Y_{s}\left(b,a\right)$	wavelet spectrum of raw signal
$N_{n}\left(b,a\right)$	wavelet spectrum of noise signal
α	reduction factor of wavelet spectral subtraction
a	wavelet filter location parameter
b	wavelet filter scale parameter
$x_{1}$	number of divisions in time domain
$x_{2}$	threshold value for parameter to be outstanding
$x_{3}$	minimum number of outstanding parameters for window to be outstanding
A	number of outstanding windows
$A_{norm}$	normalized number of outstanding windows
$D_{f}$	number of divisions in frequency domain
$C_{p}$	penalty weight for N-R subtraction
$R_{c}$	outstanding degree of window
$L_{S}$	length of synchronized datasets
$N_{n}$	number of normal datasets
$N_{a}$	number of abnormal datasets
$N_{n-r,ij}$	number of normal datasets that make window at *i*-th row and *j*-th col outstanding in N-R spectrogram
$N_{a-r,ij}$	number of normal datasets that make window at *i*-th row and *j*-th col outstanding in A-R spectrogram
$B_{ij}$	counting number of *i*-th row and *j*-th col
